# Transcriptional profiling of intervertebral disc in a post‐traumatic early degeneration organ culture model

**DOI:** 10.1002/jsp2.1146

**Published:** 2021-04-08

**Authors:** Shangbin Cui, Zhiyu Zhou, Xu Chen, Fuxin Wei, R. Geoff Richards, Mauro Alini, Sibylle Grad, Zhen Li

**Affiliations:** ^1^ AO Research Institute Davos Davos Switzerland; ^2^ Guangdong Provincial Key Laboratory of Orthopedics and Traumatology The First Affiliated Hospital of Sun Yat‐sen University Guangzhou China; ^3^ The Seventh Affiliated Hospital of Sun Yat‐sen University Shenzhen China

**Keywords:** biomarker, immunofluorescence, intervertebral disc, organ culture, RNA sequencing, traumatic

## Abstract

**Introduction:**

The goal of this study is to characterize transcriptome changes and gene regulation networks in an organ culture system that mimics early post‐traumatic intervertebral disc (IVD) degeneration.

**Methods:**

To mimic a traumatic insult, bovine caudal IVDs underwent one strike loading. The control group was cultured under physiological loading. At 24 hours after one strike or physiological loading, RNA was extracted from nucleus pulposus (NP) and annulus fibrosus (AF) tissue. High throughput next generation RNA sequencing was performed to identify differentially expressed genes (DEGs) between the one strike loading group and the control group. Gene Ontology (GO) functional and Kyoto Encyclopedia of Genes and Genomes analyses were performed to analyze DEGs and pathways. Protein‐protein interaction (PPI) network was analyzed with cytoscape software. DEGs were verified using qRT‐PCR. Degenerated human IVD tissue was collected for immunofluorescence staining to verify the expression of DEGs in human disc tissue.

**Results:**

One strike loading resulted in significant gene expression changes compared with physiological loading. In total 253 DEGs were found in NP tissue and 208 DEGs in AF tissue. Many of the highly dysregulated genes have known functions in disc degeneration and extracellular matrix (ECM) homeostasis. ACTB, ACTG, PFN1, MYL12B in NP tissue and FGF1, SPP1 in AF tissue were verified by qRT‐PCR and immunofluorescence imaging. The identified DEGs were involved in focal adhesion, ECM‐receptor interaction, PI3K‐AKT, and cytokine‐cytokine receptor interaction pathways. Three clusters of PPI networks were identified. GO enrichment revealed that these DEGs were mainly involved in inflammatory response, the ECM and growth factor signaling and protein folding biological process.

**Conclusion:**

Our study revealed different DEGs, pathways, biological process and PPI networks involved in post‐traumatic IVD degeneration. These findings will advance the understanding of the pathogenesis of IVD degeneration, and help to identify novel biomarkers for the disease diagnosis.

## Graphical abstract

This study characterized global gene expression changes and gene regulation networks in a disc organ culture system that mimics early post‐traumatic intervertebral disc degeneration. Molecules dysregulated by one strike loading were identified and may serve as biomarkers for early disc degeneration caused by detrimental mechanical loading.

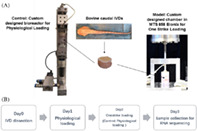

## 1 INTRODUCTION

Low back pain (LBP) is a significant healthcare concern worldwide, which leads to major economic and social burdens that affect millions of people and is a leading reason for seeking medical attention with the lifetime prevalence approaching 84%.^1^ Degeneration of the intervertebral disc (IVD) is a major contributing factor to chronic LBP. The presence of individuals over the age of 50 with evidence of intervertebral disc degeneration (IVDD) approaches 90%, correlating with lifetime prevalence of LBP.^2^ IVDD is characterized by increased imbalance between extracellular matrix (ECM) breakdown and matrix synthesis.^3^, ^4^ This imbalance results in dehydration of the central nucleus pulposus (NP), reduced proteoglycan content, decreased cellularity, diminished endplate density, and disruption of the annulus fibrosus (AF).^5^ Environmental exposures, as well as genetic and epigenetic factors have been associated with disc degeneration and altered ECM synthesis in disc tissues.^6^ Many complex and interdependent factors, including aging, decreased nutrient supply, unphysiological loading (e.g., overloading), have been implicated in the initiation and progression of IVDD. Among these factors, unphysiological mechanical loading is generally thought to play a crucial role in the process of IVDD, which is associated with ECM degradation and cellular loss.^7^ Different methods have been developed to induce IVDD in animals including gene silencing, application of supraphysiological loading, and disc injury. The advantage of using ex vivo models to study IVDD is the ability to carefully control confounding environmental variables, which may differ between individual animals such as nutrition and the loading environment.^8^ IVD whole organ culture with different degeneration initiating factors can mimic various phenotypes of disc degeneration.^9^

The ability to treat symptomatic IVDD effectively is hindered by an incomplete understanding of the biological processes that control IVD development, function, and disease. Consequently, the clinical management of IVD pathologies remains very limited, with no options at present for early intervention or predictive patient screening. Therefore, there is a need for an improved understanding of the molecular pathophysiological mechanisms underlying IVDD, which is essential for diagnosis and the development of novel therapeutic approaches. Recently, the molecular basis of IVDD has received increased attention in research, which has substantially improved the understanding of the biology underlying this process. Studies investigating the molecular changes associated with the pathophysiology of IVDD have established criteria to distinguish degenerative IVDs.^4^, ^10^ Studies evaluating transcriptome data using microarrays have provided us with an initial understanding of the molecular mechanisms underlying disc biology.^11^, ^12^ Furthermore, high‐throughput screening of human patient samples may identify potential biomarkers of IVDD, leading to more precise diagnostic criteria, classification of disease progression, and prognosis.

Genes work in synergy with each other to perform biological functions. They simultaneously interact with multiple genes and trigger a variety of changes that lead to diverse reactions. The functional annotation of regulated genes, using Gene Ontology (GO), has enabled the identification of severely affected groups of genes that correlate with the disease phenotypes.^13^ Therefore, the analysis of the gene expression profile by applying bioinformatics methods remains necessary to identify differentially expressed genes (DEGs) in IVDD and further elucidate the potential pathogenesis mechanisms of the disease.

High impact loading is one of the major causes leading to disc herniation.^14^, ^15^ The herniation may not occur right after the one strike overload, but years after. Therefore, the one strike overload model was selected to mimic the post‐traumatic IVD degeneration.^16^, ^17^, ^18^ Previously, we have established an IVDD model using one strike loading to mimic the post‐traumatic pathological changes in whole organ cultured IVD. A single hyperphysiological mechanical compression applied to healthy bovine IVDs caused significant drop of cell viability, altered the mRNA expression in the IVD, and induced ECM degradation.^19^ The present study aimed to identify the DEGs induced by one strike loading and further analyze their functions and pathways associated with the progression of IVDD by utilizing bioinformatics methods. To achieve this objective, we evaluated RNA profiles of NP and AF cells from the bovine IVD organ culture model utilizing high throughput next generation RNA sequencing. Furthermore, immunofluorescent staining was used to validate the expression of selected DEGs in human degenerated IVD tissue.

## 2 MATERIALS AND METHODS

### 2.1 IVD organ culture model and RNA extraction

#### 2.1.1 Dissection and culture of bovine IVDs with endplates

Caudal bovine IVDs (n = 8) were obtained from 4 to 10 months old animals as previously described.^20^, ^21^ Briefly, after removal of the soft tissues, IVDs comprising endplates were harvested using a band‐saw. The endplates (1–2 mm thickness) were rinsed with Ringer solution using the Pulsavac jet‐lavage system (Zimmer, Warsaw, Indiana). The discs were further incubated in phosphate buffered saline (PBS) solution with 1000 units/mL penicillin and 1000 μg/mL streptomycin for 10 min. The cleaned discs were transferred to 6 well‐plates and cultured in an incubator at 37°C, 85% humidity and 5% CO_2_ until the next day. The culture medium was composed of Dulbecco's modified Eagle medium (DMEM) containing 4.5 g/L glucose and supplied with 10% fetal calf serum, 100 units/mL penicillin, 100 μg/mL streptomycin (all products from Gibco, Basel, Switzerland) and 0.1% Primocin (Invitrogen, San Diego, California). Discs (mean IVD height 9.47 ± 1.27 mm, mean IVD diameter 15.96 ± 1.75 mm) were randomly assigned to one of two groups: one strike loading model (n = 8/group), physiological loading control (n = 8/group).

#### 2.1.2 Loading protocol

**FIGURE 1:**
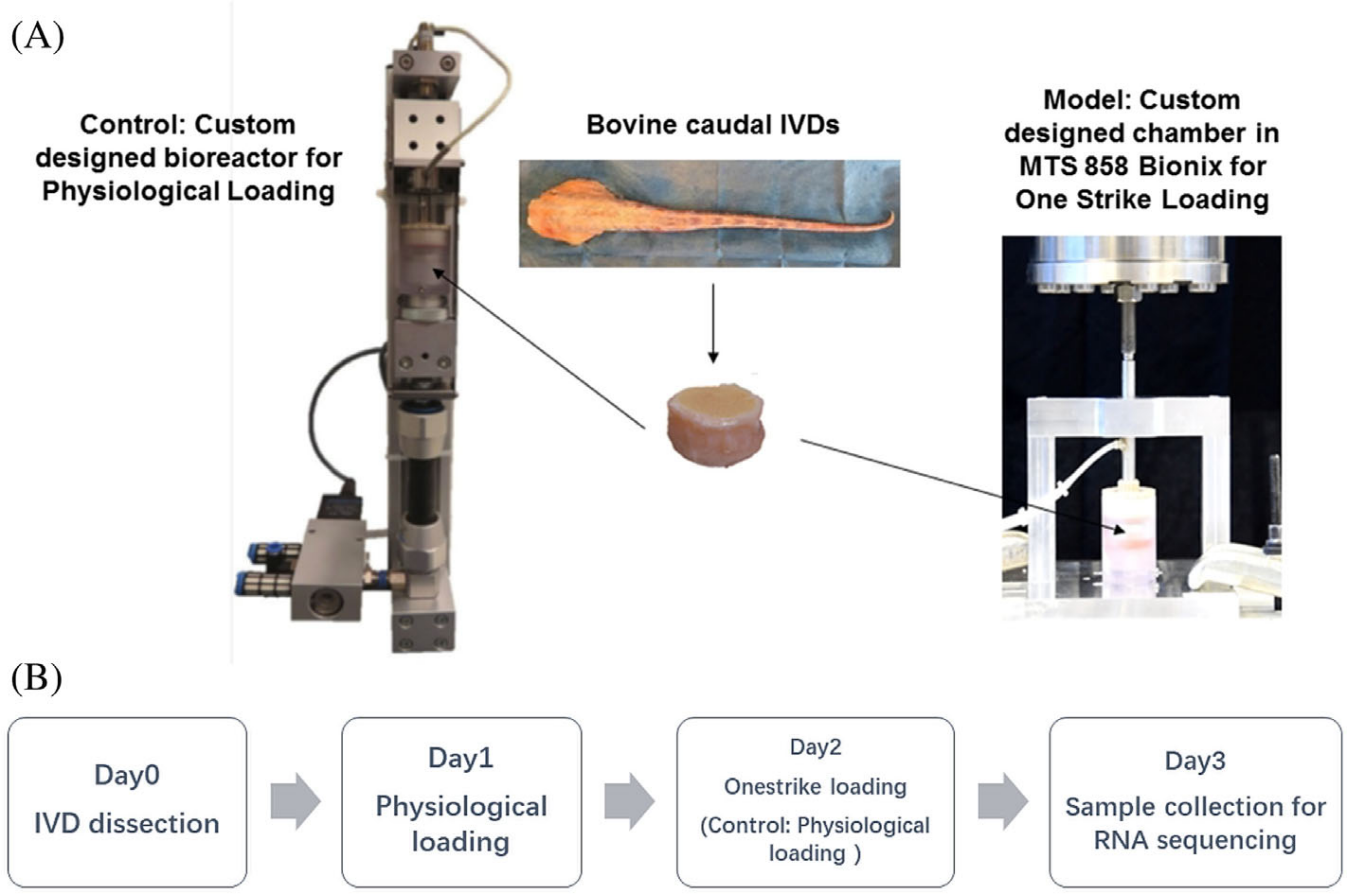
The custom designed bioreactor for physiological loading, and MTS machine with custom designed chamber for one strike loading on bovine IVDs (A). The experiment design flow chart (B). Figure is adapted from Reference 19 with permission under the terms of CC BY‐NC‐ND license

After dissection on day 0, the IVDs were cultured under physiological loading at 0.02–0.2 MPa, 0.2 Hz, 2 hours per day within a custom designed bioreactor on day 1.^9^, ^21^, ^22^ In our previous study, we found that this loading protocol caused a disc height loss around 10%,^21^ which is similar to the physiological disc height loss in human lumbar discs after diurnal activities.^23^ The bioreactor was maintained in an incubator at 37°C, 85% humidity and 5% CO_2_. On day 2, IVDs from the one strike loading group were subjected to one strike loading in a custom designed chamber or physiological loading within the bioreactor; the physiological loading control group received physiological loading. One strike loading was applied using a Mini Bionix 858 MTS machine. Isolated discs were held under a mild load of 10N for 3 min to reach steady contact with the load cell. Discs were then subjected to a single compression at 50% of disc height at a velocity of 10 mm/s. The average peak stress achieved in the one strike loading model group was 35.461 ± 8.274 MPa. The culture medium was composed of DMEM containing 4.5 g/L glucose and supplied with 2% fetal calf serum, 1% ITS+ Premix (Discovery Labware, Inc., Bedford, USA), 50 μg/mL ascorbate‐2‐phosphate (Sigma‐Aldrich, St. Louis), nonessential amino acids, 100 units/mL penicillin, 100 μg/mL streptomycin, and 0.1% Primocin. The medium of the IVD samples was changed twice per day, before and after loading. The IVDs from both groups were cultured within the custom designed chambers during loading and kept under free swelling in 6‐well plates in‐between of loading (Figure 1).

#### 2.1.3 RNA extraction

Disc tissue was collected on day 3 for gene expression measurement. Cartilaginous endplates of each IVD were removed, and NP was harvested using a biopsy punch and the outer AF tissue was harvested from the outer region of AF using a scalpel blade.

For RNA extraction, approximately 150 mg of each NP and AF tissue was used. Tissue samples were digested in 2 mg/mL pronase for 1 h at 37°C, flash frozen, pulverized in liquid nitrogen, and homogenized using a TissueLyser (Qiagen, Venlo, Netherlands).^24^ Total RNA was extracted with TRI Reagent (Molecular Research Center).

### 2.2 RNA sequencing

In total 12 samples were performed with RNA sequencing, which included 3 NP samples and 3 AF samples from the control group, and 3 NP samples and 3 AF samples from the one strike model group. Library preparation and RNAseq were performed at the Genomics Core Facility “KFB ‐ Center of Excellence for Fluorescent Bioanalytics” (University of Regensburg, Regensburg, Germany; www.kfb-regensburg.de). Library preparation and RNAseq were carried out as described in the NuGen Trio RNA‐Seq User Guide (NuGen Technologies, Inc., San Carlos, California), the Illumina HiSeq 1000 System User Guide (Illumina, Inc., San Diego, California), and the KAPA Library Quantification Kit ‐ Illumina/ABI Prism User Guide (Kapa Biosystems, Inc., Woburn, Massachusetts).

In brief, 10 ng of total RNA was used to generate amplified cDNA with priming at the 3′ end as well as randomly throughout the transcriptome. Next, libraries were prepared by fragmentation of the ds‐cDNA, end repair to generate blunt ends, and adaptor ligation. Finally, the rRNA AnyDeplete probe mix was added to facilitate degradation of unwanted ribosomal RNA transcripts. The remaining library molecules were then PCR amplified (6 cycles) to produce Illumina‐compatible libraries, which were quantified using the KAPA SYBR FAST ABI Prism Library Quantification Kit. Equimolar amounts of each library were pooled, and the pools were used for cluster generation on the cBot with the Illumina TruSeq PE Cluster Kit v3. The sequencing run was performed on an HiSeq 1000 instrument using the indexed, 2 × 100 cycles paired end (PE) protocol and the TruSeq SBS v3 Reagents according to the Illumina HiSeq 1000 System User Guide. Image analysis and base calling resulted in .bcl files, which were converted into .fastq files with the bcl2fastq v2.18 software.

### 2.3 qRT‐PCR validation

The DEGs expression in one strike model group and control group from the deep sequencing data analysis was validated by qRT‐PCR from another five samples per tissue type per group. Reverse transcription was performed with SuperScript VILO cDNA Synthesis Kit (Life Technologies, Carlsbad, California). Quantitative real‐time polymerase chain reaction was performed using the Quant Studio 6 Flex instrument (Life Technologies). The Assay ID of primers and probes (Thermo Fisher Scientific Inc.) for the measured genes are shown in Table 1. Comparative Ct method was performed for relative quantification of target mRNA with Ribosomal protein large P0 (RPLP0) as endogenous control.^20^

**TABLE 1:** Primers and probes used for real time qRT‐PCR

Gene	Assay ID
ACTB	Bt03279174_g1
ACTG	Bt03225204_m1
PFN1	Bt03219406_g1
MYLK	Bt04307621_m1
MYL12B	Bt03257882_m1
FGF1	Bt03212662_m1
IBSP	Bt03212719_m1
SPP1	Bt03213107_m1
COL9A2	Ch04673877_g1
COL11A2	Bt03229863_m1
RPLP0	Bt03218086_m1

### 2.4 Preprocessing and differential analysis

Raw data from RNA sequencing were converted into a recognizable format with the package affy of R (http://bioconductor.org/packages/release/bioc/html/affy.html,version 1.54.0), and missing values were then inferred by a method based on k‐nearest neighbors (k‐NN). Following background correction and data normalization with the median method, differential analysis between one strike loading samples and controls was performed using limma package (version 3.32.5). For statistical analysis and the assessment of differential expression, an empirical Bayes method from limma was employed to moderate the standard errors of the estimated log2‐fold changes. The basic statistic used for significance analysis is the moderated *t*‐statistic, which is computed for each probe and for each contrast. Moderated *t*‐statistics lead to *P*‐values in the same way as ordinary *t*‐statistics, except that the degrees of freedom are increased, reflecting the great reliability associated with the smoothed standard errors. Limma functions top table and decide tests were used to summarize the results of the linear model, perform hypothesis tests and adjust the *P*‐values for multiple testing. The results obtained include log2‐fold changes, standard errors, *t*‐statistics, and *P*‐values.^25^ Log|2(fold change)| >1 and *P* <0.05 were set as the cut‐offs to screen out DEGs.

### 2.5 GO functional enrichment analysis of DEGs

In order to understand the importance of genes and to identify disturbed biological functions in IVDD, we preformed GO classification, which included the following categories: biological process, cellular component and molecular function. GO functional enrichment analysis was performed for DEGs using the Database for Annotation, Visualization and Integrated Discovery (DAVID: https://david.ncifcrf.gov/) with a threshold of *P* <0.05.^26^ DAVID provides a comprehensive set of functional annotation tools for the investigation of the biological context of large lists of genes.

### 2.6 Pathway enrichment analysis of DEGs

The Kyoto encyclopedia of Genes and Genomes (KEGG: http://www.genome.jp/kegg) database is a collection of online databases consisting of genomes, enzymatic pathways, and biological chemicals. In our study, KEGG pathway enrichment analysis was performed to determine the function of DEGs using KOBAS 2.0 with a threshold of *P* <.05. KOBAS 2.0 is a web server that provides a comprehensive functional annotation tool. The biological pathways were associated with genes based on mapping to genes with established annotations. Statistical testing was performed to identify statistically significantly enriched pathways and diseases.^27^

### 2.7 Protein‐protein interaction network modules construction

Since one gene always acts in synergy with other partners, the protein interaction should also be studied when exploring the function of one gene and its protein. The Biological Networks Gene Ontology tool (BiNGO: http://www.psb.ugent.be/cbd/papers/BiNGO/), as a Cytoscape plugin, is an open‐source Java tool to determine which GO terms are significantly overrepresented in a set of genes.^28^ BiNGO was used to perform functional annotation for the network with a threshold of adjusted *P* <.05 based on hypergeometric distribution. Cluster analysis was performed using the Molecular Complex Detection Algorithm (MCODE) plugin (version: 1.5.1) in Cytoscape^28^, ^29^ to identify the clustering modules in the protein‐protein interaction (PPI) network. Significant modules were identified according to the clustering score using the following criteria: “Degree cutoff = 2,” “node score cutoff = 0.2,” “Haircut = true,” “Fluff = false,” “k core = 2,” and “max depth = 100.” The clustering modules having high node scores and connectivity degrees were considered as biologically significant clusters.

### 2.8 Immunofluorescence staining of human IVD tissue

To validate protein expression of the selected DEGs in human IVD tissue, immunofluorescencent staining of degenerated human IVD sample was performed. Human IVD samples were collected from 7 patients (3 males and 4 females, average age was 45.0 ± 3.7 years old, average course of disease was 2.3 ± 1.8 years) diagnosed with lumbar disc herniation. The samples were graded as Pfirrmann Grade IV based on the MRI scanning. The study was approved by the Ethic Committee from the seventh affiliated hospital of Sun Yat‐sen University, Shenzhen, China.

Human IVD tissues were fixed in 4% paraformaldehyde for 24 hours and dehydrated in graded sucrose solutions. Tissues were embedded in optimal cutting temperature (OCT) compound and then cut into 10 μm thick cryosections. The sections were permeabilized in PBS containing 0.3% Triton X‐100 (BioFroxx) for 30 minutes, and then blocked in PBS containing 5% bovine serum albumin (BSA) (BioFroxx) and 0.1% Triton X‐100 for 1 hour. Subsequently, sections were incubated overnight at 4°C in dilutions of antibodies against COL11A2 (1:100, MA5‐27738, Thermofisher), MYLK (1:100, PA5‐79716, Thermofisher), OPN (1:1000, ab8448, Abcam), ACTG (1:500, ab123034, Abcam), PFN1 (1:100, ab124904, Abcam), MYL12B (1:250, ab137063, Abcam), ACTB (1:100, P60709, Cell Signaling), OPN (1:50, sc‐73 630, Santa Cruz), COL9A2 (1:50, sc‐398 130, Santa Cruz), or FGF1 (1:200, 3139S, Cell Signaling). After thoroughly washing the sections with Tris‐Buffered Saline + Tween (TBST), sections were incubated with Alexa Fluor‐594 conjugated anti‐mouse secondary antibody (Jackson ImmunoResearch Inc.) at 1:300 dilution for 1 hour at room temperature, and then washed with TBST before counterstaining with DAPI (Abcam) for 5 minutes. Images were acquired using Carl Zeiss LSM880 point scanning confocal microscope.

## 3 RESULTS

### 3.1 Identification of DEGs

**FIGURE 2:**
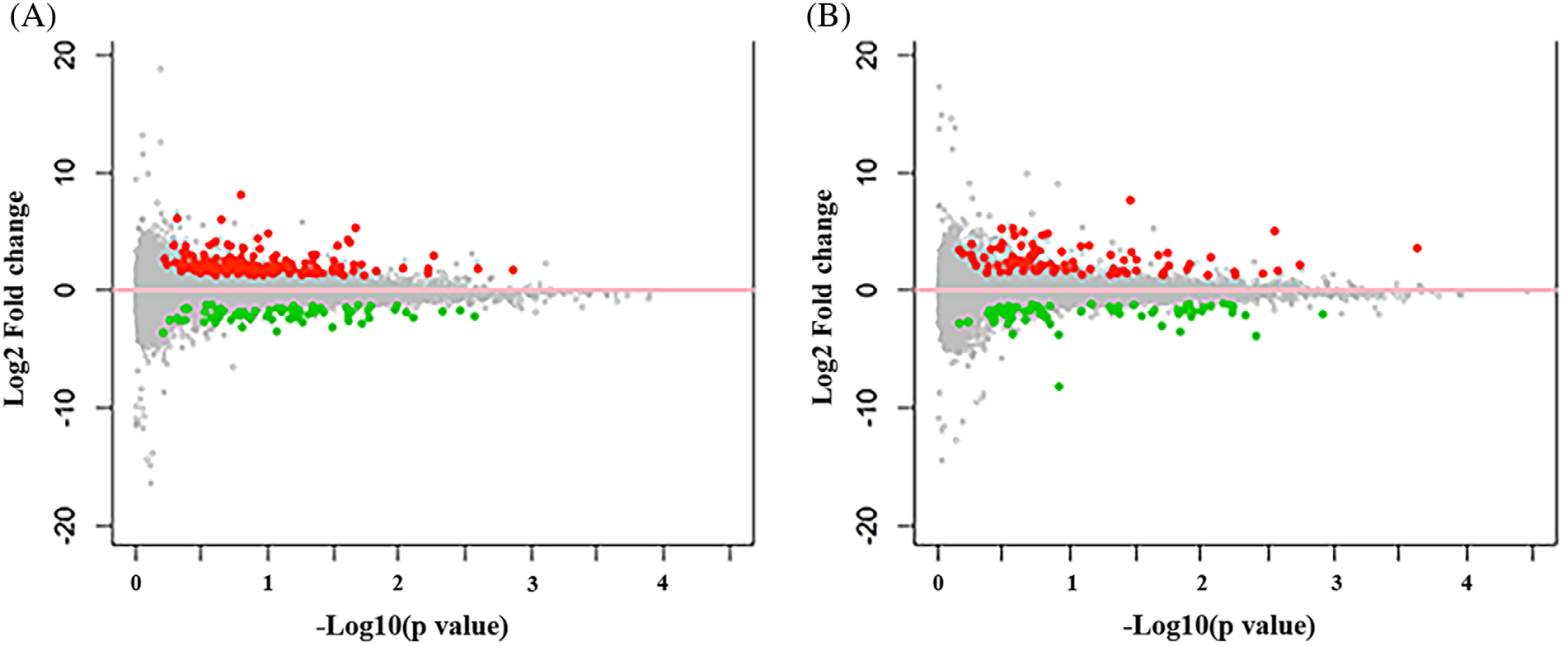
A, Volcano map of DEGs in NP tissue from one strike group compared with control group. B, Volcano map of DEGs in AF tissue from one strike group compared with control group. The red spots represent significantly upregulated DEGs, the green spots represent significantly downregulated DEGs. The number of up and down regulated DEGs were 167 and 85, respectively in NP tissue. The number of up and down regulated DEGs were 167 and 85, respectively in AF tissue. *N* = 3

Following RNA sequencing and gene expression data normalization, 167 DEGs upregulated in NP, 85 DEGs downregulated in NP, 119 DEGs upregulated in AF, and 89 DEGs downregulated in AF were identified comparing samples from one strike loading group with control group, as demonstrated in Figure 2. SRGAP1, ACTB, LAMC2, NT5E, SLC39A1, and so on were upregulated in NP tissue. SPARCL1, LUM, RAB3B, PEBP4, CCDC3, and so on were downregulated in NP tissue. As for the AF tissue, ZNF202, FGF1, IBSP, SPP1, COL9A2, and so on were upregulated. CCL20, SELP, CXCL6, CCR7, OR13C3, and so on were downregulated. The 10 genes most highly upregulated and downregulated in the NP and AF tissue by one strike loading are shown in Tables 2 and 3.

**TABLE 2:** The 10 genes most highly upregulated and downregulated in the NP tissue of one strike loading group compared with control group

Gene symbol	Gene description	Expression (log2 FC)	*q* value
SRGAP1	SLIT‐ROBO Rho GTPase activating protein 1	6.11006	0.00752755
ACTB	Actin beta	6.11006	0.0497655
LAMC2	Laminin subunit gamma 2	4.82383	0.00752755
NT5E	5′‐nucleotidase ecto	4.29025	0.0335318
SLC39A1	Solute carrier family 39 member 10	4.11986	0.00752755
TGFB2	Transforming growth factor beta 2	4.06713	0.00752755
DCLK1	Doublecortin like kinase 1	3.89048	0.00752755
EDNRA	Endothelin receptor type A	3.85173	0.00752755
KCNQ5	Potassium voltage‐gated channel subfamily Q member 5	3.80757	0.0318759
PCDH18	Protocadherin 18	3.75432	0.00752755
SPARCL1	SPARC like 1	−3.49287	0.00752755
LUM	Lumican	−3.1196	0.00752755
RAB3B	RAB3B, member RAS oncogene family	−3.11691	0.0318759
PEBP4	Phosphatidylethanolamine binding protein 4	−2.80628	0.00752755
CCDC3	Coiled‐coil domain containing 3	−2.75002	0.036885
PLAT	Plasminogen activator, tissue type	−2.69019	0.00752755
KRT18	Keratin 18	−2.57957	0.0128296
RNF165	Ring finger protein 165	−2.52701	0.0223545
ACER2	Alkaline ceramidase 2	−2.51472	0.00752755
NRN1	Neuritin 1	−2.48998	0.0318759

*Note*: Expression level is shown as log2 fold‐change (log2 FC) compared with control group. *Q*‐value is a *P*‐value that has been adjusted for the False Discovery Rate (FDR). The FDR is the proportion of false positives you can expect to get from a test.

**TABLE 3:** The 10 genes most highly upregulated and downregulated in the AF tissue of one strike loading group compared with control group

Gene symbol	Gene description	Expression (log2 FC)	*q* value
ZNF202	Zinc finger protein 202	5.25824	0.0327713
FGF1	Fibroblast growth factor 1	5.2507	0.00706143
IBSP	Integrin binding sialoprotein	5.11863	0.0409847
SPP1	Secreted phosphoprotein 1	5.03232	0.00706143
COL9A2	Collagen type IX alpha 2 chain	4.95712	0.00706143
LRRNA	Mitochondrial large ribosomal RNA	4.8385	0.00706143
ACP5	Acid phosphatase 5, tartrate resistant	4.70526	0.00706143
R3HDML	R3H domain containing like	4.63876	0.0248809
COL11A2	Collagen type XI alpha 2 chain	3.9921	0.0248809
MOXD1	Monooxygenase DBH like 1	3.80951	0.00706143
CCL20	C—C motif chemokine ligand 20	−3.84307	0.00706143
SELP	Selectin P	−3.71518	0.00706143
CXCL6	C—X—C motif chemokine ligand 6	−3.51951	0.00706143
CCR7	C—C motif chemokine receptor 7	−2.8038	0.00706143
OR13C3	Olfactory receptor family 13 subfamily C member 3	−2.77869	0.0298284
GJA5	Gap junction protein alpha 5	−2.50576	0.00706143
IFIH1	Interferon induced with helicase C domain 1	−2.3716	0.0409847
PDK4	Pyruvate dehydrogenase kinase 4	−2.33045	0.00706143
GRO1	Chemokine (C—X—C motif) ligand 1	−2.20667	0.00706143
PODXL2	Podocalyxin like 2	−2.18998	0.00706143

*Note*: Expression level is shown as log2 fold‐change (log2 FC) compared with control group. *Q*‐value is a *P*‐value that has been adjusted for the False Discovery Rate (FDR). The FDR is the proportion of false positives you can expect to get from a test.

### 3.2 GO functional enrichment and pathway enrichment analysis of DEGs

**FIGURE 3:**
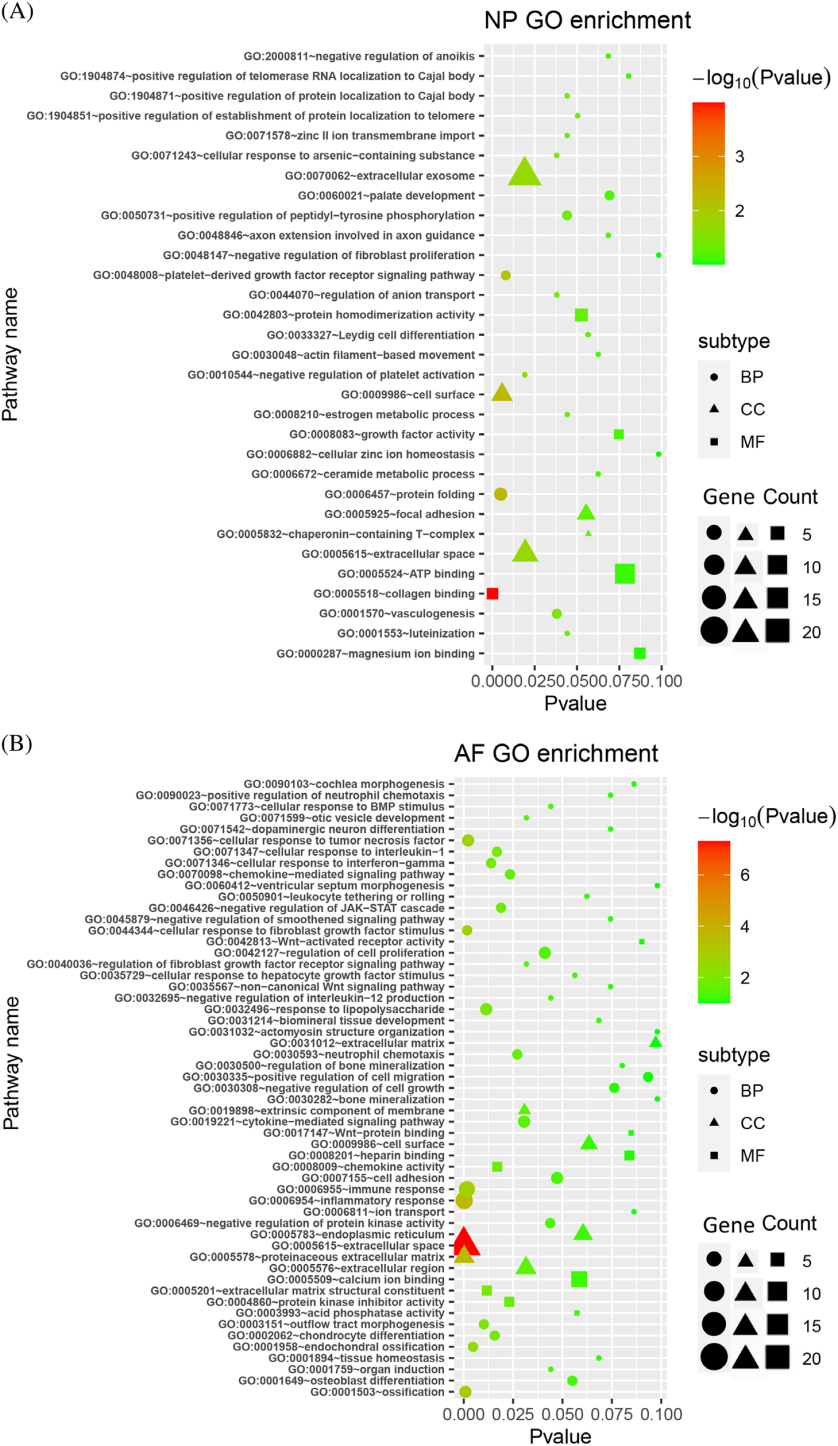
GO analysis of DEGs in NP tissue (A) and AF tissue (B). GO analysis includes Biological Process (BP), Cellular Component (CC), and Molecular Function (MF). The size of the symbols represents the numbers of genes involved in the pathway

Function annotation of the DEGs, and the clustering groups were obtained by GO function enrichment analysis. A total of 31 and 55 GO terms were enriched among the DEGs in NP and AF compared with control group, respectively (Figure 3). The phenotypic and epigenetic changes associated with disc degeneration are complex, reflecting the interplay of many distinct processes. The GO analysis suggested that many different processes were altered at early stage of disc degeneration induced by a single high impact loading. In AF tissue, the DEGs were related to ossification, immune response, cellular response to fibroblast growth factor, and tumor necrosis factor biological processes. As for the cell component, the DEGs were mainly related with the ECM and extracellular space. We also found that the DEGs may play a role in ECM structural constituent and chemokine activity. In NP tissue, the DEGs may contribute to platelet‐derived growth factor receptor signaling pathway and vasculogenesis biological process. In addition, the DEGs may be involved in cell surface, extracellular exosome, extracellular space, and focal adhesion cell component. As for the molecular function, the DEGs were related with collagen binding and protein homodimerization activity.

### 3.3 KEGG pathway enrichment analysis

**FIGURE 4:**
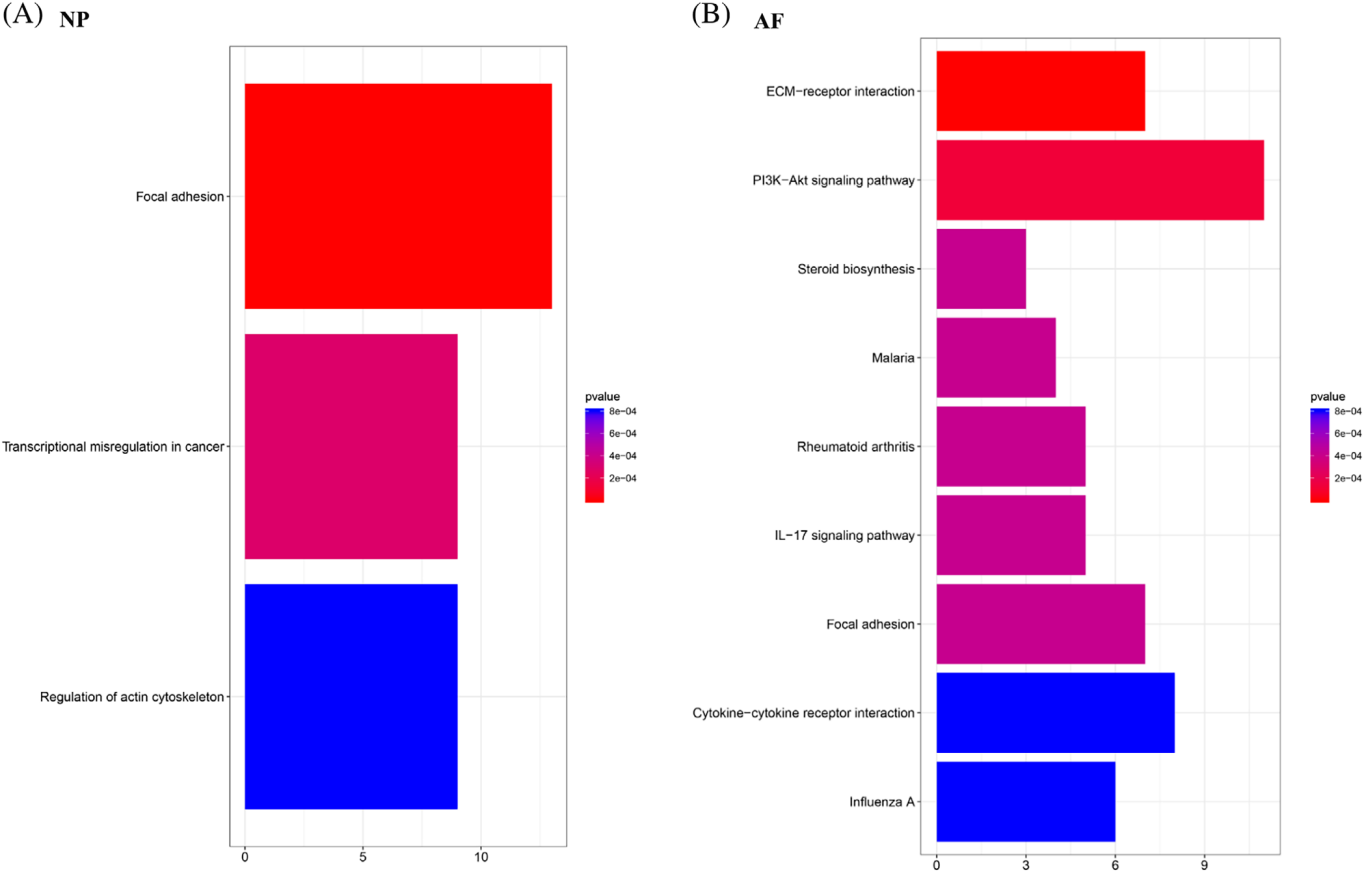
KEGG pathway enrichment in NP tissue (A) and AF tissue (B). The numbers on *X*‐axis represent the number of enriched genes in each pathway. The colors in the pathways represent different *p* value

A total of 3 and 9 pathways were disclosed for DEGs by KEGG pathway enrichment analysis in NP and AF, respectively (Figure 4). Focal adhesion pathway was dysregulated by one strike loading in both NP and AF tissue compared to the control group. The KEGG analysis showed that in the ECM‐receptor interaction pathway the related genes are COL2A, COL9A, CHAD, SPPA, IBSP, and ITGA10; in the PI3K‐Akt signaling pathway, the related genes are FGF1, IGFBP3, LAMC2, CCL9, CCL2, CCL19, CCL27, CHAD, SPP1, IBSP, GNG2, and DDIT4; in the focal adhesion pathway, the related genes are LAMC2, ITGA5, PDGFA, PDGFRA, VEGFC, FLNC, MYL12B, MYLK ACTB, ACTG, and CCND1; in regulation of actin cytoskeleton pathway, the related genes include FGF11, PGDFA, PGDFRA, ITGA1, ITGA5, ACTB, ACTG, PFN1, MYLK, and MYL12B.

### 3.4 PPI network modules

**FIGURE 5:**
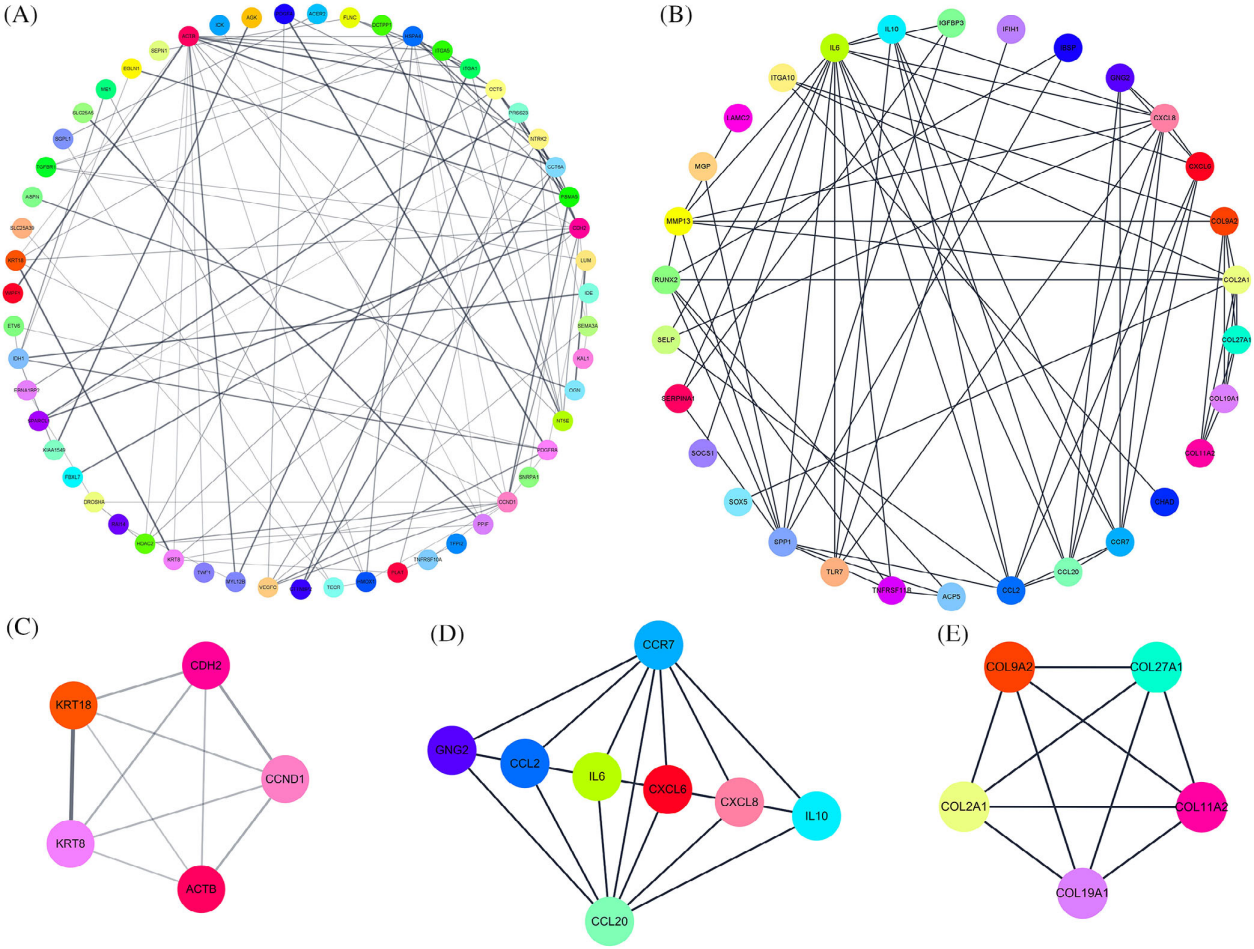
Genes related to protein‐protein networks were identified by String. Hubgenes from the PPI network were identified with the MCODE algorithm. (A) and (B) represent the PPI networks dysregulated in NP tissue and AF tissue, respectively. (C) (clustering score = 5) is the top PPI network of hubgenes extracted from (A). D, (clustering score = 6.857), E, (clustering score = 5) are the top PPI networks of hubgenes extracted from (B). MCODE, molecular complex detection; PPI, protein‐protein interaction

To reveal associations among genes within the most enriched network module, the Cytoscape Web plugin was used to visualize the input genes in a network graph. STRING (http://string-db.org) was used to analyze the gene related protein interaction network. The names of genes related to PPI network are shown in Figure 5. KRT8, KRT18, CDH2, and CCND1 were identified as a subset of hub genes in the PPI network in NP tissue, which were related to aging and proliferation. IL6, CXCL6, CCR7, and CCL20 were identified as a subset of hub genes in AF tissue which play an important role in the inflammatory response of the IVD. COL2A1, COL9A2, COL11A2, COL19A1, and COL27A1 were identified as a subset of hub genes in AF tissue related to ECM composition.

### 3.5 Validation of DEGs by real‐time qRT‐PCR

**FIGURE 6:**
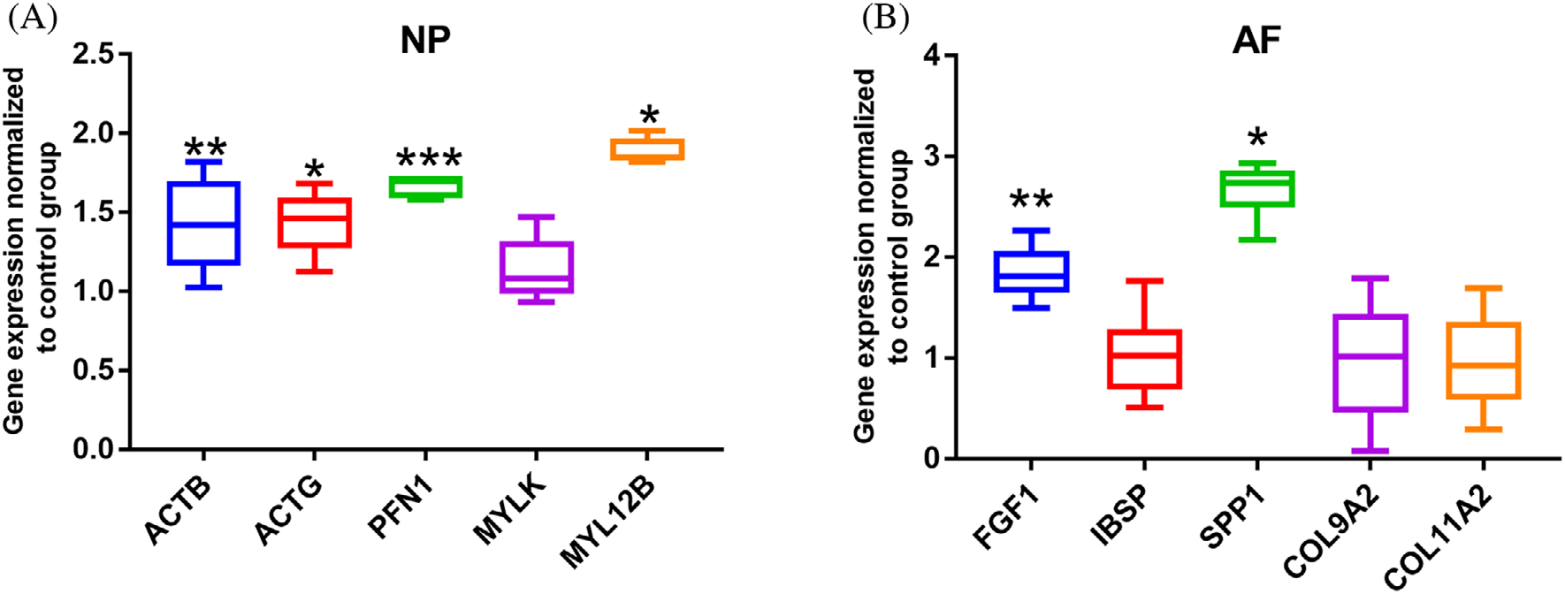
Gene expression levels of selected DEGs in one strike model group normalized to control group. n = 5, ^*^ *P* <.05, ^**^ *P* <.01, ^***^ *P* <.001, one strike model group vs control group

Confirmation of gene sequencing data with qRT‐PCR was performed for selected genes from the DEGs. These genes are related to ECM‐receptor interaction pathway, DI3K‐AKT pathway, Focal adhesion pathway and Regulation of actin cytoskeleton pathway. Five DEGs dysregulated in NP tissue and five DEGs dysregulated in AF tissue were selected for the qRT‐PCR validation. The results showed that ACTB, ACTG, PFN1, and MYL12B in NP tissue were significantly upregulated by one strike loading compared with control group. In AF tissue, FGF1 and SPP1 were significantly higher expressed in one strike model group compared with control group (Figure 6, *P* <0.05). The qRT‐PCR results confirmed the findings of the gene sequencing study.

### 3.6 Immunofluorescence staining of human IVD samples

**FIGURE 7:**
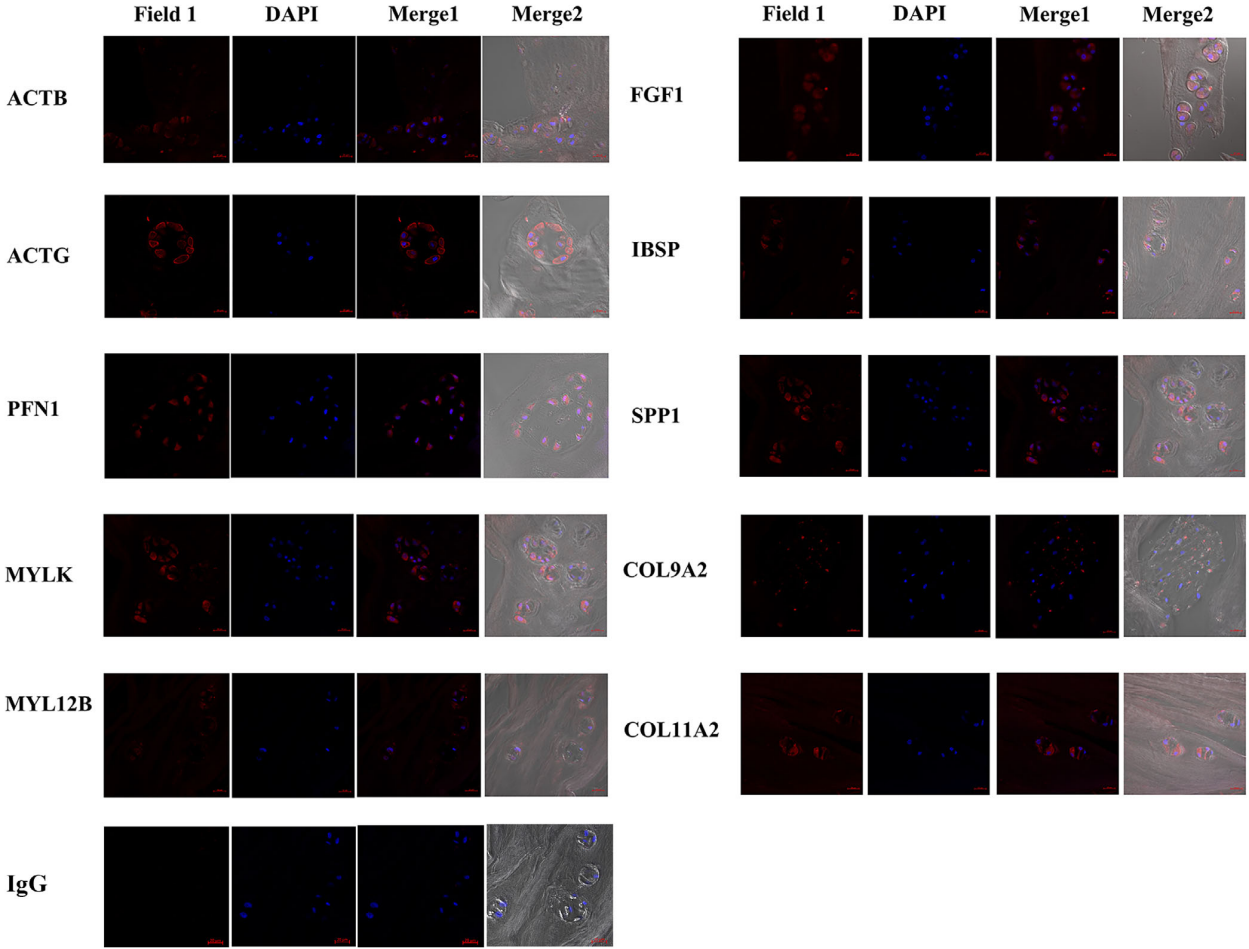
Immunofluorescence staining of ACTB, ACTG, PFN1, MYLK, MYL12B, FGF1, IBSP, SPP1, COL11A2, and COL9A2 in human degenerated IVD tissue. Scale bar = 20 μm. DAPI, 4,6‐diamino‐2‐phenyl indole (DAPI) field; Field 1, red fluorescent field stained with different antibodies; Merge1, Field1 merged with DAPI field; Merge2, bright field merged with Merge1. Representative images from N = 7 patients

To validate the protein expression of selected DEGs in human degenerated IVD samples, immunofluorescent staining was performed. All the sections showed positive staining of ACTB, ACTG, PFN1, MYLK, MYL12B, FGF1, IBSP, SPP1, COL11A2, and COL9A2 in the degenerated human IVD tissues which had a Pfirrmann grade of IV (Figure 7). The immunofluorescent signal was strong in cytoplasm stained with ACTG, PFN1, MYLK, FGF1, SPP1, and COL11A2 antibodies. ACTB, MYL12B, IBSP, and COL9A2 only showed weak positive staining signal.

## 4 DISCUSSION

Over decades of research, increasing attention has been paid to the molecular mechanisms underlying IVDD. Notable improvements in the knowledge of the pathological processes have been achieved,^29^ with multifactorial bioprocesses proven to contribute to IVDD.^30^ However, despite years of investigation, the pathogenesis underlying the initiation and progression of IVDD remains poorly understood and requires further investigation. The emergence of bioinformatics methods has accelerated the progress of research on the mechanisms of human diseases. Kazezian et al compared microarray profiles from annulus fibrous tissue of degenerative and nondegenerative discs.^31^ The result revealed that 238 DEGs was dysregulated in various cellular functions including cell proliferation and inflammatory response.^31^ Guo et al^32^ used microarray to compare the gene expression profiles between patients with IVD degeneration and control. A total 93 and 114 DEGs were identified in NP and AF tissue, respectively. GO analysis showed that the DEGs may be involved in various processes, including cell adhesion, biological adhesion, and ECM organization.^32^ KEGG analysis showed that the DEGs was involved in focal adhesion and the p53 signaling pathway.^32^ Wang et al^33^ compared the microarray data between the degenerated human IVD samples with nondegenerative samples. The GO analysis showed that the GO term cytoskeleton organization, neuron projection development, and neuron development was significantly dysregulated. The KEGG analysis showed that the cytokine‐cytokine receptor interaction, regulation of actin cytoskeleton and axon guidance pathway were also significantly dysregulated.^33^ Tang et al^34^ compared the gene expression profile between three degenerative human IVD samples and three nondegenerative samples. The GO analysis showed that the cell‐matrix adhesion, cell‐substrate adhesion and blood vessel development were significantly dysregulated.^34^ In the present study, deep RNA sequencing data of pathologically and physiologically treated tissue samples from bovine IVD were compared. The DEGs were screened and their functions were further analyzed to reveal the gene expression patterns in NP and AF after high impact loading and to identify key pathways induced. In accordance with previous studies, the ECM receptor interaction pathway, PI3K‐Akt signaling pathway, focal adhesion pathway, and actin cytoskeleton pathway were dysregulated in one strike loading induced IVD degeneration progress.

In the present study, FGF1, SPP1, ACTB, ACTG, PFN1, and MYL12B were significantly upregulated by one strike loading compared to control group, as indicated by sequencing analysis and confirmed by PCR results. In agreement with recent studies, these identified DEGs may play important roles in the pathobiology of post‐traumatic IVDD. The family of fibroblast growth factors (FGFs) is widely known to be involved in a variety of developmental and pathological processes in the human body. FGF1 gene is expressed in chondrocytes from articular cartilage of osteoarthritis patients.^35^ FGF1 molecule overexpressed in articular cartilage has shown to strongly activate the MMP13 gene, which encodes a proteinase to degrade cartilaginous ECM.^36^ FGF1 is also known to be involved in cellular movement, cellular growth and proliferation, cellular development and cell cycle in the IVD.^8^ SPP1, also known as osteopontin (OPN), is an extracellular structural protein secreted by various types of cells. SPP1 could activate the integrin proteins in NP cells and has close connections with COL11A1, COL3A1, and COL2A1.^37^ Since integrins are a class of cell adhesion molecules that regulate interactions between cells and their surrounding matrix, activation of integrin alpha (ITGA) will assist NP cells to interact with the ECM, specifically collagen molecules and then to potentially restore the IVD function.^38^ Marfia et al reported greater expression of SPP1 in degenerative IVD compared to herniated IVD, and SPP1 was only detected in degenerative IVD tissue. It is speculated that SPP1/OPN may be a marker for the severity of disc degeneration.^39^ The actin cytoskeleton is essential for the proper functioning of many cellular processes, including maintenance of the cell shape, chemotaxis, cell movement, adhesion, transport of cellular organelles, mitosis, replication, transcription, and even DNA repair.^40^, ^41^ Studies had identified that β‐actin predominates in stress fibers while γ‐actin is enriched at the leading edge of cell surface.^42^ Studies with immature bovine IVDs showed that AF cells respond to cyclic tensile strain (10%, 1 Hz) with increase in the expression of β‐actin and β‐tubulin at both the transcriptional and protein level.^43^

Cell‐matrix adhesion has essential roles in a number of important biological processes, including cell motility, proliferation and differentiation, and the regulation of gene expression and cell survival; at contact points between the cell and ECM, specialized structures termed focal adhesions are formed.^44^ The response of cells to loading, and the specific mechanotransduction pathways mediating responses to load, are dependent on cell morphology, cell‐cell interactions, and cell‐ECM interactions. Loading of cells can act directly on cytoskeleton networks by promoting F‐actin reorganization, which can act as a mechanism for transducing mechanical stimuli to the nucleus.^43^, ^45^ Actin cytoskeletal remodeling is largely mediated by the RhoA/ROCK signaling pathway, which is one important part of focal adhesion pathway also identified by our research. The RhoA/ROCK signaling pathway is involved in many responses such as cell migration, polarization, stress fiber, and adhesion formation.^46^ RhoA/ROCK signaling is also altered in response to mechanical stimuli, which creates complex interactions between morphological and biomechanical changes.^47^ Supporting these findings, Guo et al^32^ identified 35 genes that were differentially expressed in NP and AF of degenerated IVDs compared with nondegenerated tissues by analyzing microarray data. Their results suggested that collagen type VI α 2 chain, integrin binding sialoprotein, RAP1A, and FOXF2 play important roles in the focal adhesion signaling pathway associated with IVDD.

An inflammatory response is thought to initiate IVDD, and pro‐inflammatory molecules, secreted by IVD cells are considered to mediate IVDD.^48^ These cytokines trigger a range of pathogenic responses by the disc cells that can promote autophagy, senescence and apoptosis.^6^, ^49^, ^50^ The resulting imbalance between catabolic and anabolic responses leads to degeneration, as well as herniation and radicular pain.^48^ In the present study, IL6, CXCL6, CCR7, and CCL20 were identified as a subset of hub genes in the PPI network which was constructed in the AF tissue. Accordingly, it may be speculated that this combination of genes has a key role in the immediate response of AF cells to mechanical stress. IL6 plays a critical role in IVDD.^51^ Herniated IVDs have been reported to spontaneously produce IL6.^52^ Binding of IL‐6 to IL‐6R/gp130 complex primarily signals through Ras and PI3K pathways^53^ which were identified by our KEGG enrichment analysis.^54^, ^55^ Grad et al evaluated serum levels of the chemokines C—C motif ligand 6 (CXCL6, also known as granulocyte chemotactic protein 2), and observed higher systemic levels of CXCL6 in patients with disc degeneration compared with healthy controls.^56^ Philips et al investigated the expression of cytokine and chemokine associated genes in NP cells from degenerated human IVDs and found that CCL2 and CXCL8 expression was detected more frequently in degenerate samples compared to nondegenerate samples. The protein expression of CCL2 and CXCL8 increased concordant with histological degenerative tissue changes.^57^ CCL2 levels in degenerated and herniated canine IVDs were also found to be significantly higher compared with nondegenerated and nonherniated IVDs.^58^ CCL20 was expressed in degenerated human NP cells and CCL20‐CCR6 system was involved in the trafficking of IL‐17 producing T‐cell subet (Th17) to degenerated IVD tissues.^59^ CCR7+ and CD163+ cells increased with degeneration grade of human IVD samples and were significantly enhanced in IVD regions with structural irregularities and defects.^60^

The ECM is a component of all mammalian tissues, and is a network that consists predominantly of the fibrous protein collagen, elastin and fibronectin. In addition to a structural function, the ECM exhibits a number of other roles. As a major component of the cellular microenvironment, it affects various cell behaviors, which include proliferation, adhesion and migration, and also regulates cell differentiation and death.^61^ ECM composition is particularly heterogeneous and dynamic and abnormal ECM dynamics may lead to dysregulated cell proliferation, cell death failure and loss of cell differentiation. During the onset of one strike loading induced IVD degeneration, catabolic factors, cytokines and inflammatory factors are produced by the native disc cells. In our previous study, the gene expressions of MMP1, MMP3, ADAMTS4, ADAMTS5, CXCL5, and IL‐6 were up‐regulated in NP tissue 1 day after one strike loading, and the gene expressions of MMP1, MMP13, and ADAMTS5 were upregulated in AF tissue 8 days after one strike loading. The glycosaminoglycans released in the culture medium from the one strike model was significantly higher than the control group.^19^ The results showed the disturbance of the balance between anabolism and catabolism of the ECM during the IVD degeneration.^19^ Disc degeneration also affects the synthesis of ECM proteins. Collagen and aggrecan are important structural proteins and proteoglycans within the disc, which must resist high tensile loads to maintain the disc shape. These ECM protein genes are often dysregulated during IVDD. For example, disc degeneration is typically associated with an upregulation of collagen I that leads to a loss of compliance and hardening of the NP. Downregulation of collagen II and upregulation of collagen I in NP is eventually seen in a degenerative IVD. One explanation for these findings is that the initial reparative attempts by the IVD restore ECM expression patterns, which only later become irrevocably altered.^8^ In the present study, COL2A1, COL9A2, COL11A2, COL19A1, and COL27A1 were identified as a subset of hub genes in the PPI network which was affected by one strike loading in the AF tissue. GO analysis also indicated that collagen genes were enriched in ECM structural constituent. Zhu et al^62^ cultured rabbit IVD samples under static loading and found that immunohistochemical staining intensity and gene expression of COL2A1 were significantly enhanced. Polymorphisms of the COL9 and COL11 genes contribute to the progression of degenerative lumbar disc stenosis.^63^ Videman et al^64^ documented five different polymorphisms in COL11 genes that were significantly associated with signs of disc degeneration such as reduced disc signal and disc bulging. Solovieva et al^65^ found that a sequence variation in intron 9 of COL11A2 was associated with an increased risk of disc bulges compared with those people without polymorphism. Findings from our results and literatures suggest that changes in collagen occur in IVD after abnormal loading, which may contribute to further deterioration of IVD degeneration.

In the present study, KRT8, KRT18, CDH2, and CCND1 were identified as a subset of hub genes in the PPI network which was constructed in the NP tissue. The cytokeratin (KRT) gene family is responsible for the transcription of keratins, a type of fibrous protein. Microarray study on human NP cells showed that KRT8 and KRT18 were significantly decreased in degenerate human NP cells.^11^ Recent consensus has identified a panel of markers specific for the healthy juvenile NP cell phenotype, including *N*‐cadherin (CDH2), changes in expression of which is associated with aging and degeneration.^66^, ^67^ Hwang et al^68^ investigated the role of CDH2 mediated cell contacts in regulating human NP cell morphology and phenotype. The results showed that promotion of cell‐cell interactions via CDH2 signaling leads to enhanced biosynthesis and NP marker expression. Holguin et al^69^ used a mice IVD model to investigate the relationship of β‐Catenin, transcription factor of Wnt signaling, and IVD degeneration. The results showed that deletion of β‐Catenin reduced expression of proliferation marker CCND1 by 18%.

Biological treatments are designed based on the biological changes occurring in IVD degeneration. The mechanism underlying IVD degeneration is complex and correlates with changes at molecular level. With an enhanced understanding of the disease from a molecular biology perspective, novel strategies are likely to emerge to prevent and treat degenerative IVDs. To the best of our knowledge, this is the first study utilizing a bovine organ culture model and RNA deep sequencing to investigate the transcriptomic changes responsible for the early events induced by mechanical overloading of the IVD. Moreover, this reproducible model allows for the real‐time evaluation of catabolic and inflammatory processes which may lead to degradation of the ECM and ultimately to loss of the biomechanical properties of the IVD.

There are several limitations in our study. First, the main focus was to investigate the mechanism of early degeneration change induced by mechanical loading. However, IVDD can also be induced by other factors such as nutrient insufficiency, cell senescence, and these factors may have a significant influence on the biomolecular signaling events within IVD degeneration. Second, follow up of the subsequent degeneration mechanism was not investigated in the current study. Third, immunofluorescence staining was performed on degenerative human IVD samples to verify the protein expression of selected DEGs. However, this is only preliminary investigation without healthy human IVD tissue as control group. Further research on the expression level of the DEGs in healthy control and human IVD tissue at different degeneration grades will be needed to verify their potential as biomarker or therapeutic target for post‐traumatic early IVD degeneration. Finally, only a set of genes that were already known to be related with IVD degeneration were validated by qRT‐PCR and immunofluorescence staining.

## 5 CONCLUSIONS

In conclusion, the present study investigated the early transcriptome changes after single high impact load of the IVD via bioinformatics analysis. A total of 252 DEGs were identified in the impacted NP and 208 DEGs were identified in the impacted AF. The combination of ACTB, ACTG, PFN1, MYL12B, and SPP1 may serve as biomarkers for early responses to mechanical overloading. These DEGs may be involved in focal adhesion, ECM‐receptor interaction, PI3K‐AKT and cytokine‐cytokine receptor interaction pathways during the early phase of IVD degeneration caused by mechanical loading. GO enrichment revealed that these DEGs were mainly involved in inflammatory response, the ECM, and growth factor signaling and protein folding processes. These molecules are potential novel targets of therapeutic strategy for the treatment or prevention of post‐traumatic IVD degeneration. The findings shed new light on the pathophysiology of IVD degeneration and may guide rational treatments, including gene therapy, cell therapy, and tissue engineering approaches for IVD degeneration and LBP.

## CONFLICT OF INTEREST

The authors declare no conflict of interest. The funders had no role in the design of the study; in the collection, analyses, or interpretation of data; in the writing of the manuscript, or in the decision to publish the results.

## AUTHOR CONTRIBUTIONS

**Zhiyu Zhou** and **Zhen Li**: Conceptualization. **Zhiyu Zhou**, **Shangbin Cui**, **Xu Chen**, **Fuxin Wei**, and **Zhen Li**: Methodology. **Zhiyu Zhou**, **Shangbin Cui**, **Xu Chen**, and **Zhen Li**: Formal analysis. **Zhiyu Zhou, Shangbin Cui**, **Fuxin Wei**, and **Xu Chen**: investigation. **Fuxin Wei**, **R. Geoff Richards**, **Mauro Alini**, **Sibylle Grad**, and **Zhen Li**: Resources. **Shangbin Cui**: Writing—original draft preparation. **Zhiyu Zhou, Shangbin Cui**, **R. Geoff Richards**, **Mauro Alini**, **Sibylle Grad**, and **Zhen Li**: Writing—review and editing. **Shangbin Cui** and **Zhen Li**: Supervision. **Zhen Li**: Project administration. **Zhiyu Zhou**, **R. Geoff Richards**, **Mauro Alini**, **Sibylle Grad**, and **Zhen Li**: Funding acquisition.

**DATA AVAILABILITY STATEMENT**

The raw sequencing data are deposited in NCBI, accession number PRJNA659042.
